# PhosphoPredict: A bioinformatics tool for prediction of human kinase-specific phosphorylation substrates and sites by integrating heterogeneous feature selection

**DOI:** 10.1038/s41598-017-07199-4

**Published:** 2017-07-31

**Authors:** Jiangning Song, Huilin Wang, Jiawei Wang, André Leier, Tatiana Marquez-Lago, Bingjiao Yang, Ziding Zhang, Tatsuya Akutsu, Geoffrey I. Webb, Roger J. Daly

**Affiliations:** 1 Biomedicine Discovery Institute and Department of Biochemistry and Molecular Biology, Monash University, Melbourne, VIC 3800 Australia; 2 Monash Centre for Data Science, Faculty of Information Technology, Monash University, Melbourne, VIC 3800 Australia; 3 Department of Chemical Biology, College of Chemistry and Chemical Engineering, Xiamen University, Xiamen, Fujian 361005 China; 4 Biomedicine Discovery Institute and Department of Microbiology, Monash University, Melbourne, VIC 3800 Australia; 5 Informatics Institute and Department of Genetics, School of Medicine, University of Alabama at Birmingham, Birmingham, AL USA; 6 College of Mechanical Engineering, Yanshan University, Qinhuangdao, 066004 China; 7 State Key Laboratory of Agrobiotechnology, College of Biological Sciences, China Agricultural University, Beijing, 100193 China; 8 Bioinformatics Center, Institute for Chemical Research, Kyoto University, Kyoto, 611-0011 Japan

## Abstract

Protein phosphorylation is a major form of post-translational modification (PTM) that regulates diverse cellular processes. *In silico* methods for phosphorylation site prediction can provide a useful and complementary strategy for complete phosphoproteome annotation. Here, we present a novel bioinformatics tool, PhosphoPredict, that combines protein sequence and functional features to predict kinase-specific substrates and their associated phosphorylation sites for 12 human kinases and kinase families, including ATM, CDKs, GSK-3, MAPKs, PKA, PKB, PKC, and SRC. To elucidate critical determinants, we identified feature subsets that were most informative and relevant for predicting substrate specificity for each individual kinase family. Extensive benchmarking experiments based on both five-fold cross-validation and independent tests indicated that the performance of PhosphoPredict is competitive with that of several other popular prediction tools, including KinasePhos, PPSP, GPS, and Musite. We found that combining protein functional and sequence features significantly improves phosphorylation site prediction performance across all kinases. Application of PhosphoPredict to the entire human proteome identified 150 to 800 potential phosphorylation substrates for each of the 12 kinases or kinase families. PhosphoPredict significantly extends the bioinformatics portfolio for kinase function analysis and will facilitate high-throughput identification of kinase-specific phosphorylation sites, thereby contributing to both basic and translational research programs.

## Introduction

Eukaryotic proteins are typically subjected to various post-translational modifications (PTMs) in order to enable proper and specific functioning. Among the more than 200 different types of PTMs that have been identified^1^, phosphorylation is one of the most prevalent types and plays a crucial role in almost every aspect of cell life, including metabolism, proliferation, differentiation, apoptosis, DNA replication, and cell division^2, 3^. Protein phosphorylation is catalyzed by a group of enzymes called kinases, which add a phosphate (PO_4_) group to serine (S), threonine (T), tyrosine (Y), or, to a lesser degree, histidine (H) residues. Additionally, phosphate moieties that exist on substrates can be removed by phosphatases. Therefore, phosphorylation is a reversible PTM, depending on the balance of kinases and phosphatases.

The human genome encodes more than 500 different protein kinases, collectively regulating a diverse range of signaling pathways and biological functions^4^. Recent data indicate that the majority of proteins in a eukaryotic cell can be phosphorylated^5^. As a regulatory mechanism, individual protein kinases can specifically recognize and target a subset of protein substrates for phosphorylation, i.e. they have distinctive substrate specificity^6^. Aberrant regulation of protein phosphorylation often results in disease. Many members of the human protein kinase family are implicated in cancer, reflecting alteration or dysregulation at the level of the gene, mRNA, protein and/or PTM, and they provide clinically-validated or potential targets for personalized cancer treatment^7, 8^. Therefore, identification and characterization of kinases and their specific phosphorylation sites in the proteome is a critical first step towards a complete understanding of protein-kinase-regulated signaling pathways, and their impact in health and disease.

Owing to the recent development of large-scale high-throughput mass spectrometry techniques, experimentally-verified phosphorylation data have rapidly accumulated^7–10^. For example, Sharma *et al*. describe ultradeep characterization of the phosphoproteome, detecting phosphorylation of ~75% of cellular proteins^5^. The Mann group has now moved MS phosphoproteome analysis to a high-throughput and systems-wide scale. They have recently developed a scalable phosphoproteomics platform which enables rapid quantification of hundreds of phosphoproteomes with more than 10,000 sites^9^. Despite these recent technological advances, it is likely that a significant number of phosphorylation sites remain unidentified, and upstream kinases for many phosphorylation events are unknown. Therefore, computational approaches capable of identifying phosphorylation sites and their cognate kinases complement experimental efforts and may provide a powerful additional strategy for whole-proteome annotation. With the increasing availability of sequenced genomic data for various organisms, comprehensive prediction of kinase/substrate pairs is becoming more advantageous and useful for proteome annotation and hypothesis-driven experimental design.

To date, more than a dozen tools have been developed for phosphorylation site prediction. These can be categorized into three main classes: simple consensus pattern-based approaches, sequence similarity-based clustering methods, and more advanced machine-learning algorithms. ELM^11^, PROSITE^12^, and HPRD^13, 14^ are examples from the first category. These approaches depend upon the presence of an exact motif surrounding the phosphorylation site. Sequence similarity-based methods such as PostMod^15^ and PSEA^16^ are designed to give a high score to a query peptide that has a high similarity score with known phosphorylation peptides, using sequence similarity measures like the BLOSUM62 matrix^17^. Since definitions of consensus patterns are often based on limited data, the performance of such methods in predicting phosphorylation sites is poorer than that observed from more advanced methods. Additionally, consensus pattern-based methods can only provide binary prediction outputs. Accordingly, such methods are not suitable for large-scale analysis and probabilistic scoring schemes^18^.

In the last decade, a number of machine learning-based approaches have been employed to address the task of phosphorylation site prediction. These include artificial neural networks (ANN)^19^ (NetPhosK^20, 21^), hidden Markov models (HMM)^22^ (KinasePhos^23, 24^), Bayesian decision theory (BDT)^25^ (PPSP^26^), support vector machines^27^ (PredPhospho^28^, PPRED^29^, and Musite^30, 31^), and conditional random fields (CRFs) (CRPhos^32^). Since machine learning-based methods can learn the underlying rules and signatures in the data by tuning and optimizing related parameters during the model training process, their performance is usually comparable to or even better than the performance of consensus pattern-based methods.

Most current methods focus on predicting phosphorylation sites by integrating sequence and other informative information. Linding *et al*. developed a computational approach called NetworKIN to predict phosphorylation networks and assign substrate specificity, which takes into consideration the context of protein-protein interactions^33^. Benchmarking tests indicate that the NetworKIN approach can yield a 2.5-fold improvement in accuracy, while also allowing for construction of phosphorylation networks^33^. Recently, Li *et al*. proposed a more sophisticated approach for the prediction of protein phosphorylation sites, which integrates primary sequences with heterogeneous features, such as protein functional information, protein subcellular location, and protein-protein interaction information^34^. The authors investigated eight different human kinases or kinase families (ATM, CDKs, CK2, GSK-3, MAPKs, PKA, PKB, and PKC) to evaluate the contribution of functional features to the prediction of kinase-specific phosphorylation sites based on 5-fold cross-validation tests and found that functional features significantly boosted prediction performance for seven kinases, with the ATM family being the only exception^34^. More recently, Wang and colleagues developed computational approaches^35, 36^ to predict kinase-specific phosphorylation sites by combining both sequence and functional information of proteins (such as Gene Ontology and protein-protein interactions), based on random forest and support vector machines, respectively. They found that functional information is critical for determining phosphorylation sites^35, 36^.

Although significant progress has been made in predicting kinase-specific phosphorylation sites, existing approaches have a number of drawbacks. (1) Use of feature selection: Most existing tools are developed using machine-learning techniques, like SVM. However, for machine-learning models, not all features are equally important for the performance of the trained model. Inclusion of redundant features in model training reduces model performance; to remove redundant features and, consequently, improve prediction performance, feature selection is generally required. However, to this date, only a limited number of studies have adopted this strategy to gain insight into the relative significance and contributory effects of various features. (2) Incorporation of heterogeneous features: With the notable exceptions of NetworKIN^33^ and Li *et al*.^34^, most previous studies only extracted features based on the sequence environment surrounding the phosphorylation sites, but failed to take other relevant heterogeneous features into consideration. These include structural and other global features that might play a decisive role in determining a protein’s phosphorylation propensity, especially for those involved in different cellular processes or having different protein-interaction or pathway characteristics. There is an outstanding need to investigate and characterize the importance and contribution of functional features to model performance across different kinase families and examine if there exist family-specific subsets of distinct features. (3) Analysis based on enlarged datasets: While a few methods take protein functional features into account, analyses were performed on limited, outdated datasets and the quantitative contribution of such methods needs to be systematically evaluated on sufficiently large and updated datasets. Moreover, Li *et al*. did not provide either a webserver or a local tool. In summary, the next generation of computational methods needs to address the above drawbacks in order to generate more accurate models for efficient identification of kinase-specific phosphorylation sites.

In this paper, we present PhosphoPredict, a new tool developed for computational prediction of human kinase-specific phosphorylation sites. Our tool is based on the original idea of Li *et al*. to integrate heterogeneous protein functional features with sequence-derived features. However, we augmented a machine-learning algorithm, Random Forest (RF)^37^, by integrating a variety of heterogeneous features at multiple levels (sequence, structure and function) to train the kinase-specific classifiers. In particular, to improve phosphorylation site prediction performance, we integrated protein sequence-derived features and structural features together with other complementary functional features, including gene ontology (GO) terms, Kyoto Encyclopedia of Genes and Genomes (KEGG) pathways, protein-protein interactions, and protein functional domains.

In this work, we describe our tool and present a feature-importance analysis for each individual kinase family performed with the goal of identifying the most relevant and contributing features. Based on an independent test dataset, we compare the performance of PhosphoPredict with four other popular tools, including KinasePhos^23, 24^, PPSP^26^, Musite^30, 31^, and GPS^38–40^, for phosphorylation site prediction for human kinases CDKs, MAPKs, PKC, and CK2. Lastly, we present results of PhosphoPredict, here applied with 99% specificity to the entire human proteome, showing a large number of newly identified potential substrates targeted for phosphorylation. While we focus here on 12 human kinases or kinase families, namely ATM, CaM, CDKs, CK1, CK2, GRK, GSK-3, MAPKs, PKA, PKB, PKC, and SRC, it is important to note that our approach can be used to develop substrate and phosphorylation-site predictors for any kinase family not only for humans, but also for other organisms such as plants and bacteria.

## Materials and Methods

### Datasets

#### Positive dataset

Phosphorylation sites were extracted from the Phospho.ELM Database (version 9.0)^41, 42^, which is a public database of experimentally verified phosphorylation sites in eukaryotic proteins. The current release (Version 9.0) contains 8718 substrate proteins from different species covering more than 42,500 sites. In this study, we focused on human kinase-specific phosphorylation site prediction and, consequently, extracted all human phosphorylation datasets, comprising a total of 37,145 entries and 5374 human proteins. Furthermore, in order to reduce sequence redundancy in the extracted datasets and avoid potential bias in model training, we employed the same procedures as described by Li *et al*. and removed highly homologous sequences (at the 70% sequence identity) using the CD-HIT program^43^. Specifically, phosphorylation sites were extracted for each human kinase family and only the major kinase families that contained at least 50 experimental phosphorylation sites were included in the analysis. Table 1 provides a statistical summary of the kinase families included and their corresponding substrates and phosphorylation sites. Among the 12 types of protein kinases studied, CDKs and MAPKs are not single protein kinases but represent two protein kinase families. Indeed, the term MAPK comprises 14 kinases belonging to three subfamilies, the ERK, JNK and p38, and the atypical ERKs. This might raise the question whether the members of the three subfamilies differ in their consensus phosphorylation sites. However, this seems not to be the case, at least for the ERK, p38 and JNK family members^44^. In our preliminary analysis, we generated pLogos (Figure S1) of the occurrences of amino acid residue types surrounding the phosphorylation sites for each of the three kinase types. We found that they indeed share a consensus phosphorylation site recognition motif, namely XXPS/TPXX, requiring proline residues at the +1 and (to a lesser extent) −1 position (“X” denotes any amino acid residue type)^44^. Thus, it is justified to train phosphorylation site prediction models for the overall MAPK family and the use of “MAPK” is a valid category in the context of predicting potential phosphorylation substrates and sites using PhosphoPredict. In the case of CDKs, these enzymes also exhibit a preference for substrate peptides that exhibit a proline residue at the +1 position after the phosphorylated residue, but we accept that there are subtle differences in substrate selectivity amongst family members^45^. For clarity, we will refer to “CDKs” and “MAPKs” instead of “CDK” and “MAPK” throughout this paper.

**Table 1:** Statistics of human kinase-specific substrates and their phosphorylation sites, derived from the Phospho.ELM database (version 9.0).

Kinase	Number of substrate sequences	Number of phosphorylation sites
ATM (Ataxia telangiectasia mutated)	29	58
CaM (Calcium/calmodulin-dependent protein kinase)	37	62
CDKs (Cyclin-dependent kinases)	120	274
CK1 (Casein kinase 1)	20	56
CK2 (Casein kinase 2)	108	255
GRK (G protein-coupled receptor kinase)	18	73
GSK-3 (Glycogen synthase kinase 3)	32	60
MAPKs (Mitogen-activated protein kinases)	132	312
PKA (cAMP-dependent protein kinase)	138	218
PKB (Protein kinase B)	54	77
PKC (Protein kinase C)	150	308
SRC (Src-family tyrosine kinase)	63	100

**Figure 1:**
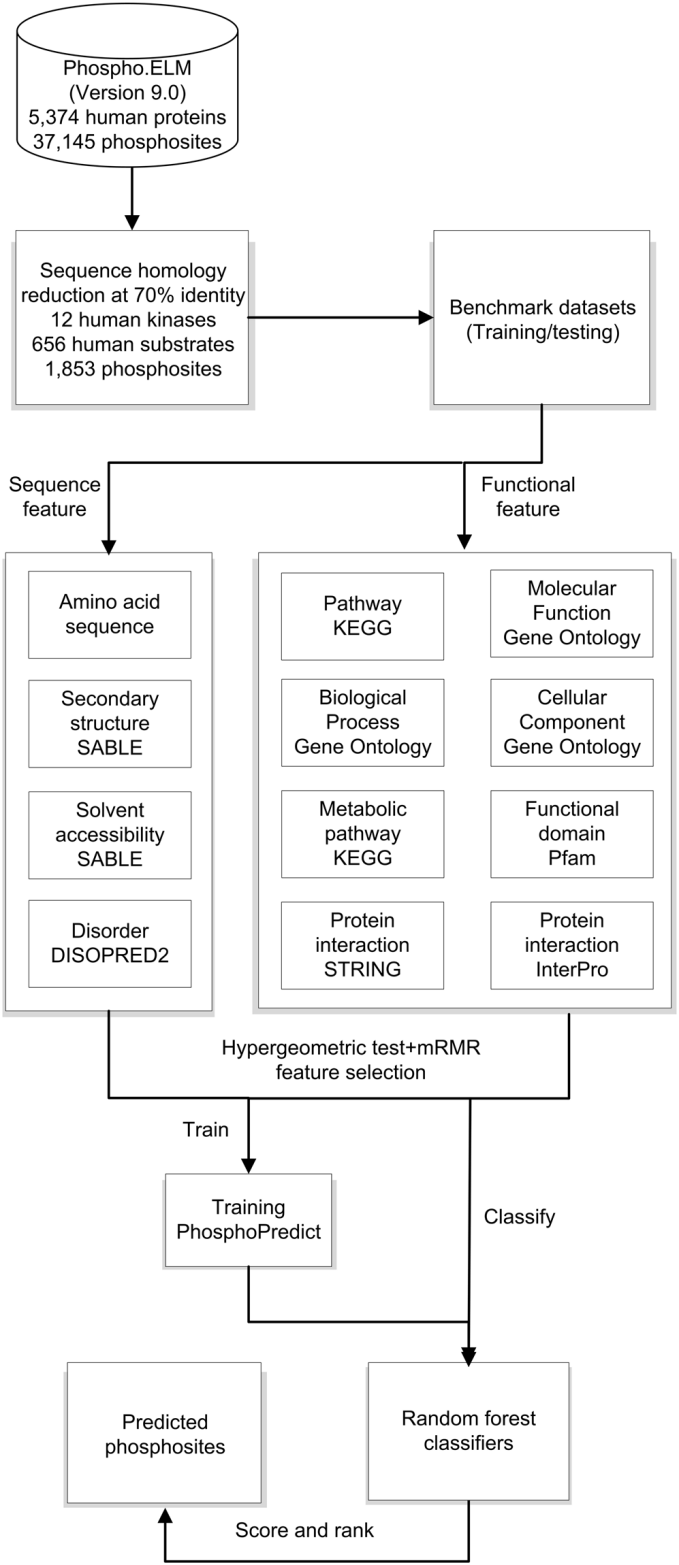
Workflow of the PhosphoPredict approach. Benchmark training/testing datasets were extracted from the Phospho.ELM database after removing sequence redundancy (70% sequence identity) using the CD-HIT program^39^. After feature selection using mRMR and statistical analysis of over-represented and under-represented feature terms using hypergeometric tests, significant sequence, structural, and functional features were extracted and used as inputs to train RF classifiers. Classifier performance was assessed using randomized 5-fold cross-validation and independent tests.

To evaluate the model performance, we prepared a benchmark dataset and two independent test datasets (See the “Independent tests” section for details). The performance of the model was evaluated using randomized 5-fold cross-validation on the benchmark dataset and validated on the two independent datasets. For each potential phosphorylation site, a local sliding window of nine residues was used, which included four amino acids in the upstream and four amino acids in the downstream regions surrounding the central residue. The workflow of our developed PhosphoPredict approach is shown in Fig. 1.

#### Background set

All human proteins were extracted from the UniProt database^46^ and used as the background protein set. The background set was used to perform statistical analysis and to identify statistically significant functional features (See detail below).

#### Background set and negative dataset

We constructed the background set by extracting all S/T/Y (serine, threonine, or tyrosine) residues from the background protein set. The negative samples were then randomly selected from the background set.

### Features

We derived a variety of different features and examined them regarding their impact on model performance. In addition to sequence-derived and functional features, we also integrated structural features, including protein secondary structure, solvent accessibility, and native disorder, which have proven useful in previous studies of phosphorylation site prediction. These features are briefly discussed in the following subsections.

#### Sequence level features

*Amino acid type*. The amino acid sequences surrounding phosphorylation sites are primary sequence features and have proven useful for phosphorylation site prediction in previous studies^34^. We encoded amino acid sequences using the 20-bit binary encoding method, wherein each amino acid was represented by a 20-dimensional binary vector composed of either zero or one elements as described previously^47, 48^. Using a sliding window comprised of nine amino acids, this led to a 20 × 9 = 180-dimensional vector.

*Predicted secondary structure*. Protein secondary structure is a powerful attribute used for predicting phosphorylation sites. However, given that known protein secondary structure information is limited, we instead predicted protein secondary structure from amino acid sequences by using SABLE^49^. Specifically, for each residue of the query sequence, SABLE outputs three kinds of secondary structure: H, E, and C, denoting alpha-helix, beta-strand, and coil, respectively. We encoded the three kinds of predicted secondary structure using a 3-bit encoding, yielding a 3 × 9 = 27-dimensional vector.

*Predicted solvent accessibility*. Solvent accessibility is also an important feature for phosphorylation site prediction^34^. The SABLE program^49^ can also be used to predict solvent accessibility from primary sequences. It provides a score from 0 to 6, representing the extent of solvent accessibility from ‘buried’ to ‘exposed’. Therefore, we used a 7-bit encoding for the predicted solvent accessibility, thus resulting in a 7 × 9 = 63-dimensional vector.

*Predicted natively-disordered region*. Disordered protein regions lack fixed tertiary structure and are either fully or partially unfolded^50^. Contrary to initial suggestions that these regions are ‘useless’, recent studies indicate that such regions are commonly involved in many biological functions^50^. For example, phosphorylation sites have been observed to be preferentially located in disordered rather than ordered regions^51, 52^. Accordingly, some studies used protein disorder information as an important feature for phosphorylation site prediction^51, 53^. We predicted the native disorder information using DISOPRED2^54^ and encoded it using a 2-bit encoding to form a 2 × 9 = 18-dimensional vector.

*Functional features*. In addition to sequence and structural features, the present study also employed functional features of proteins. These include: (1) Biological Process (BP) feature from GO^55^; (2) Cellular Component (CC) feature from GO; (3) Molecular Function (MF) feature from GO; (4) Functional domain information from InterPro^56^; (5) Pathway information from KEGG^57^; (6) Functional domains from Pfam^58^; (7) Protein-Protein Interaction (PPI) from STRING^59^.

### Over- and under-represented feature analysis by hypergeometric test

Heterogeneous functional features can be noisy and redundant, resulting in biased model training and performance assessment. Therefore, we performed a two-sided hypergeometric test for each kinase-specific substrate protein to identify over-represented and under-represented feature terms from the background protein set. The hypergeometric tests were performed using the R package^60^. The *p*-values were calculated from the hypergeometric distributions as follows:

$$p={F}_{hypergeom}(q,m,n,k)$$

where *q* represents the number of samples with the feature term in the study set, *m* represents the number of samples annotated with the feature in the background set, *n* represents the number of samples without the feature, while *k* is the number of samples in the study set.

The *p*-values were corrected by the Bonferroni correction for testing on multiple feature terms. Feature terms with a corrected *p*-value of less than 0.01 were considered significant.

After extracting all significant functional features, a simple log-odds ratio approach was originally proposed by Li *et al*.^61^ and used to calculate the final score of each protein as the log-odds ratio score as follows:

1 $$S({x}_{i})=\sum _{i=1}^{N}{\mathrm{log}}_{2}\frac{f({x}_{i})}{g({x}_{i})}$$

where *N* denotes the total number of significant functional features, *x*_*i*_ represents the value of the *i*-th feature which was measured by the functional annotations of the protein, *f(x*_*i*_ *)* represents the probability of the *i*-th feature in phosphorylated proteins from the positive training dataset, while *g(x*_*i*_ *)* represents the probability of the *i*-th feature in all proteins from the background protein set.

### Feature selection by maximum Relevance Minimum Redundancy (mRMR)

Feature selection is an important aspect in practical applications of machine learning. Many biological datasets are characterized by a large number of initial features for model training and optimization. Dealing with oversized feature sets is a challenging and formidable task, with several associated problems. Large feature sets slow down the speed of the machine learning algorithm, consume many resources, and are inefficient. Additionally, many machine learning methods suffer from reduced accuracy when dealing with large feature sets^62–64^. As a result, efficient feature selection methods are required to improve efficiency of machine learning-based classifiers and minimize classification error. Feature selection can select the most relevant and informative features by reducing the initially high-dimensional feature space to a lower, more compact one.

mRMR is a useful feature selection algorithm based on mutual information^65^. It was originally proposed by Peng *et al*.^65^ and can be downloaded from http://penglab.janelia.org/proj/mRMR/. The mRMR algorithm has been widely used in a number of feature-selection tasks by our group^66–68^ as well as others^69–71^, often in combination with step-wise feature selection, resulting in an improved performance of trained models. Importantly, mRMR is able to rank features according to both their relevance to the target classification variable and the redundancy between the features themselves. The features assigned with a higher rank by mRMR indicate that they have better trade-off between maximum relevance and minimum redundancy. We selected the top 50 features identified by mRMR as our optimal feature set.

### Model training using RF

RF is an ensemble classifier consisting of a number of decision trees. It was originally developed by Breiman^37^ and has been implemented as the RF package in R^72^. RF has several important advantages that make it suitable for our prediction task, including: (1) It performs better with high-dimensional feature inputs; (2) It runs efficiently on larger datasets; (3) It has higher efficiency in model training, given that the training process is faster than many other algorithms; (4) It can estimate what variables are more important for classification. Like many other machine-learning techniques, RF also includes model training and prediction stages. At the training stage RF grows many classification trees and selects the classification that receives the most votes from all trees, while at the prediction stage RF model performance is tested and evaluated.

### Randomized 5-fold cross-validation test

To evaluate the prediction performance of RF-based models, randomized five-fold cross-validation was used by randomly dividing the benchmark dataset into five subsets for each validation step. At each cross-validation step, four subsets were merged as the training set to train the RF model, while the remaining subset was singled out as the test set to validate the trained RF model. This procedure was repeated five times so that each subset was used in the training and then validated in the testing. To allow for a robust estimation of the model performance, this five-fold cross-validation procedure was repeated 100 times. As a result, we calculated the average of RF classifier performance measures, which are reported here.

### Independent tests

In addition to the randomized 5-fold cross-validation on the benchmark datasets, we have also assembled an independent test dataset and performed the independent test using this dataset to allow a fair and objective comparison to other tools. The independent dataset was extracted from another public database, PhosphoSitePlus^73^, by including the most recent experimental phosphorylation data and excluding those instances that had been deposited in the database Phospho.ELM^41, 42^. For brevity, this first independent dataset is referred to as “PhosPlus_set”. The prediction performances of our method, PhosphoPredict, and four other tools (PPSP, GPS, KinasPhos, and Musite) were evaluated based on this independent dataset.

In addition, we have also constructed a second independent test dataset, which has not been previously used in any of the other predictors. To construct it, we first downloaded the most-recent version of the UniProt database (2017 Version, last modified on 15 February, 2017). We then filtered out the overlapping sequences that were present in both the training dataset of PhosphoPredict and the obtained UniProt dataset. After this step, we further removed the homologous sequences in the training dataset and the resulting UniProt dataset, by applying the CD-HIT program with a sequence identity of 70%. The resulting independent test dataset is referred to as “UniProt_set”.

### Performance Assessment

We used several performance measures, including Sensitivity (SEN), Specificity (SPE), Precision (PRE), Accuracy (ACC), the Matthew’s correlation coefficient (MCC), and the area under the curve (AUC) to comprehensively evaluate the predictive performance of our method.

SEN is defined as:

2 $$SN=TP/(TP+FN)$$

SPE is defined as:

3 $$SP=TN/(TN+FP)$$

PRE is defined as:

4 $$PRE=TP/(TP+FP)$$

Overall ACC is defined as:

5 $$ACC=(TP+TN)/(TP+TN+FP+FN)$$

*F*-score is defined as:

6 $$F-score=2\times \frac{TP}{2TP+FP+FN}$$

The MCC^74^ is defined as:

7 $$MCC=\frac{TP\times TN-FP\times FN}{\sqrt{(TP+FN)\times (TP+FP)\times (TN+FN)\times (TN+FP)}}$$

where *TP* is the number of true positives, *TN* is the number of true negatives, *FP* is the number of false positives, and *FN* is the number of false negatives.

More specifically, AUC is the area under the receiver operating characteristic (ROC) curve, which is a plot of true positive rate (TPR) against false positive rate (FPR). TPR is the ratio of the number of correctly classified phosphorylation sites relative to the total number of phosphorylation sites, while FPR is the ratio of the number of correctly classified non-phosphorylation sites relative to the total number of non-phosphorylation sites. The performance of our method was evaluated using the seven measures based on both 5-fold cross-validation and independent tests.

## Results and Discussion

### The overall framework of the PhosphoPredict approach

We extracted phosphorylation substrate datasets for 12 kinase families from the Phospho.ELM database. We removed any sequence redundancy from the original datasets and subsequently trained RF-based models of phosphorylation site prediction independently for each of the 12 kinases or kinase families. The resulting set of models forms the core of PhosphoPredict. The tool not only identifies relationships between substrates and specific kinase families, but also predicts corresponding phosphorylation sites for the 12 kinase families in a kinase-specific manner. The overall framework of the PhosphoPredict approach is illustrated in Fig. 1. The four main stages in PhosphoPredict development are dataset curation, feature extraction, feature selection, and model training and performance evaluation. The first stage does not only involve curation but also dataset preprocessing. At the second stage, a variety of different features at multiple levels are calculated and extracted, including sequence features, predicted structural features, and protein functional features. At the third stage, hypergeometric tests are performed to identify over-represented and under-represented functional feature terms and the mRMR algorithm is applied to select the most relevant and important features. At the final stage, performance of RF-based predictors is assessed using both randomized 5-fold cross-validation and independent tests.

### Analysis of over-represented and under-represented functional features

**Figure 2:**
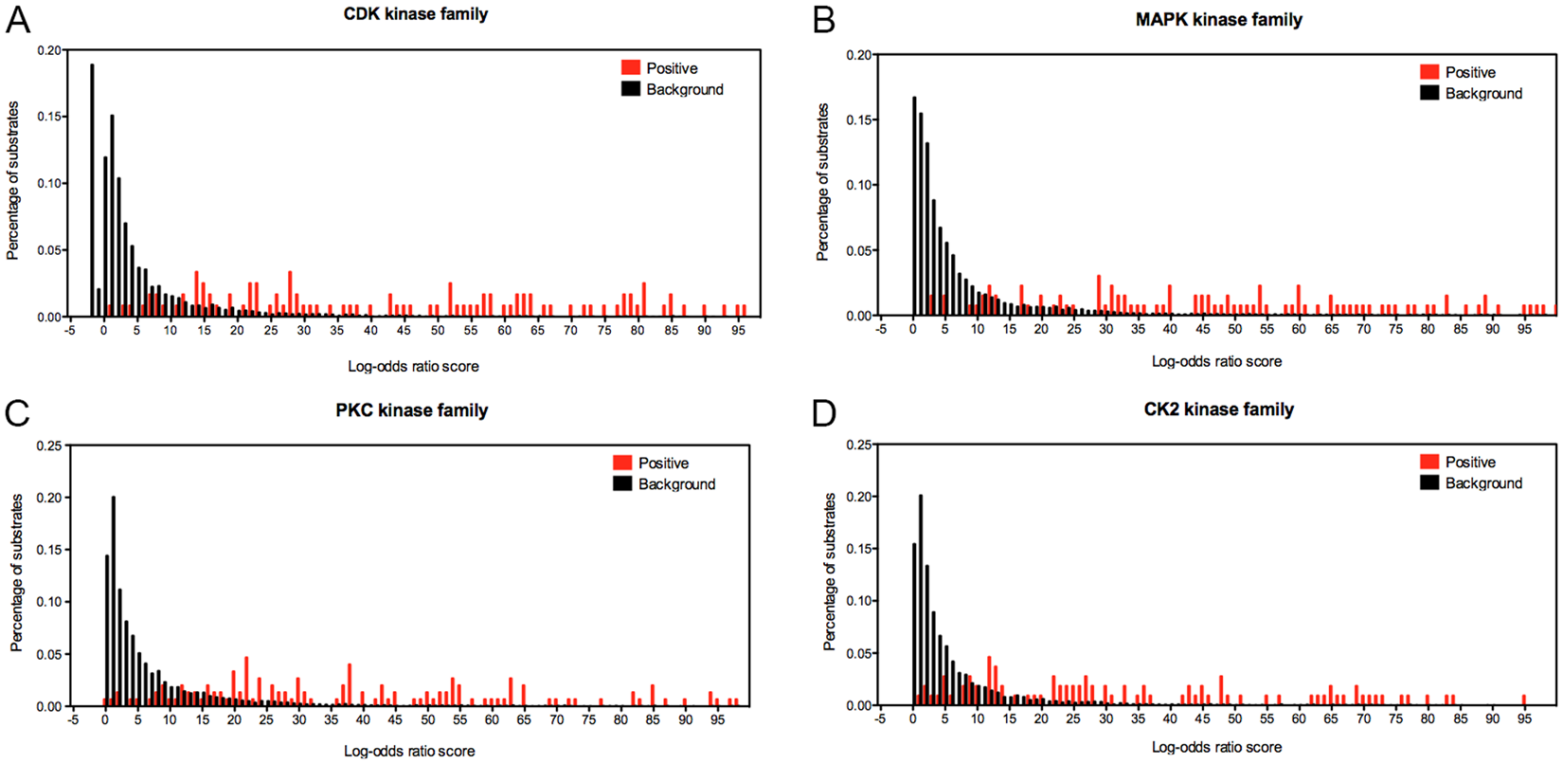
Protein substrate distributions. The distributions of the known protein substrate set (red) and the background protein set (black) for four common kinase families. The x-axis represents the log-odds ratio score, while the y-axis represents the percentage of proteins with the corresponding scores. Data represent (**A**) CDKs, (**B**) MAPKs, (**C**) PKC, and (**D**) CK2.

Protein phosphorylation is a dynamic process implicated in multiple aspects of cellular function. Determinants of phosphorylation events may comprise multifaceted functional features, such as protein-protein interactions and subcellular localization. Using a simple log-odds ratio approach^61^, we calculated the functional score of each protein as the log-odds ratio score and plotted the distributions of known phosphorylated protein substrate subsets (colored red) and background protein sets (colored black) for four common kinase families, including CDKs, MAPKs, PKC and CK2 (Fig. 2). The functional score reflects the likelihood of a corresponding protein to be phosphorylated. The higher the log-odds ratio score, the more likely a protein is to be phosphorylated. From Fig. 2, we can see that the distributions of the known protein substrate subsets (red) and background protein sets (black) are significantly different. For example, the majority of proteins in the background protein sets have scores <10, whereas proteins in the positively known substrate sets tend to have an even distribution and scores >20. These results agree with those observed by Li *et al*.^34^.

Furthermore, we performed a statistical *t*-test and calculated *p*-values to elucidate the statistical differences between functional scores of proteins in the positive substrate set versus the background set (Table 2). The most significant distribution occurs in the MAPK kinase family with a *p*-value of 5.99e-25. The least significant distribution occurs in the CK1 kinase family with a maximum *p*-value of 0.00187. These results indicate that phosphorylated substrate proteins can be discerned from the background protein set and that functional features might be helpful in distinguishing phosphorylated and non-phosphorylated proteins.

**Table 2:** Significance of functional score differences between proteins in the positive substrate set versus the background set, estimated by statistical *t*-test.

Kinase	*P*-value
ATM	1.16e-09
CaM	2.75e-09
CDKs	1.87e-22
CK1	0.00187
CK2	3.28e-12
GRK	3.37e-05
GSK-3	2.97e-06
MAPKs	5.99e-25
PKA	1.21e-23
PKB	1.99e-14
PKC	3.59e-24
SRC	2.20e-12

### Effect of functional features on predictive performance

In order to ascertain whether incorporation of significant functional features can improve prediction of phosphorylation sites, we integrated primary sequences with functional features and examined their effect on the predictive performance of the trained RF classifiers based on 5-fold cross-validation tests. All RF classifiers were trained using the default parameters and different feature combinations. Table S1 provides the results of cross-validation based on the benchmark dataset for each kind of functional group. Seven performance measures, including ACC, SEN, SPE, PRE, *F*-score, MCC, and AUC, were calculated to compare the performance of different feature combinations.

Classifier performance for all kinase families improved after combining functional features with primary sequence features. Specifically, for the GRK family, AUC increased from 0.595 (RF model trained using only primary amino acid sequence features [AA]) to 0.891 (AA + CC), 0.962 (AA + BP), 0.859 (AA + MF), 0.932 (AA + InterPro), 0.943 (AA + KEGG), and 0.901 (AA + Pfam). Additionally, there was consistent improvement in terms of other performance measures, such as ACC, *F*-score, and MCC (Table S1).

However, we noticed that when the primary sequence features were combined with other structural features, such as secondary structure (SS), solvent accessibility (SA), and native disorder (DO), the performance did not improve significantly and for certain kinase families the performance even decreased. For example, in the case of the CaM family, when primary sequence features were used in combination with structural features, AUC scores decreased from 0.822 (AA) to 0.759 (AA + SS), 0.817 (AA + SA), 0.791 (AA + DO), 0.756 (AA + SS + SA), 0.759 (AA + SS + DO), 0.783 (AA + SA + DO), and 0.770 (AA + SS + SA + DO) (Table S1). Similar trends were obtained for several other kinase families, including ATM, CK2, GSK-3, MAPK, PKB, and PKC (Table S1). These results indicate that including a large number of initial features may not coincide with improved predictive performance. Instead it can lead to performance decreases, presumably due to inclusion of noisy, irrelevant, and redundant features. Altogether, these results highlight the need to address this problem by performing feature selection to remove irrelevant features, identify more contributive features, and improve model performance.

### Feature selection results using mRMR

**Figure 3:**
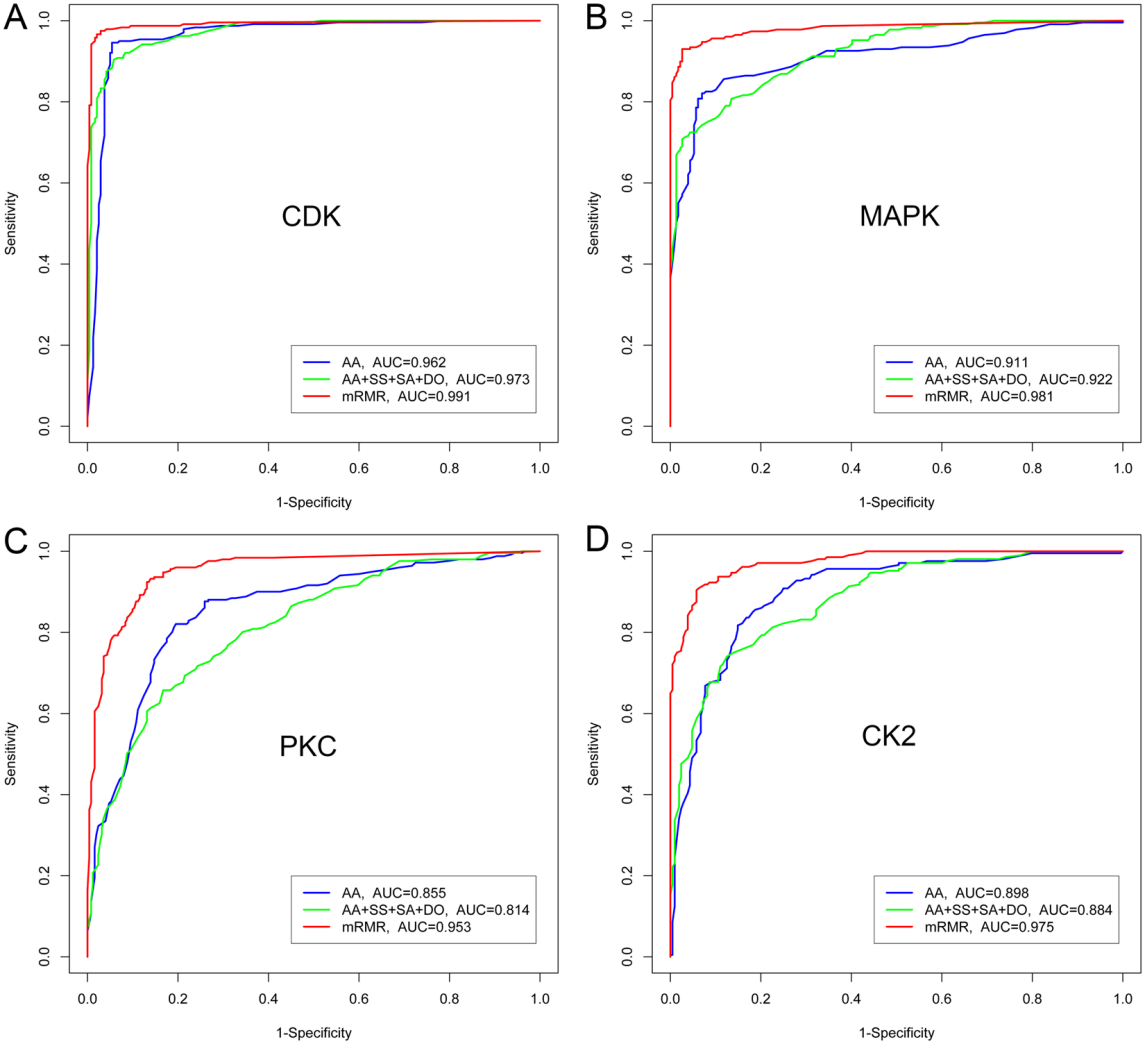
Phosphorylation site prediction. ROC curves for phosphorylation site prediction of three different sequence-encoding schemes: AA (amino acid sequence encoding), AA + SS + SA + DO (amino acid sequence + secondary structure + solvent accessibility + native disorder, without feature selection), and mRMR (mRMR feature selection based on all the extracted initial features), evaluated using 5-fold cross-validation tests on the benchmark datasets. Data represent (**A**) CDKs, (**B**) MAPKs, (**C**) PKC, and (**D**) CK2.

A protein’s set of features is represented via a 5698-dimensional vector. It describes various heterogeneous features, which are complex, noisy, and redundant. To identify the most relevant features critical for phosphorylation site prediction, we employed the mRMR method to select optimal feature subsets. Importantly, mRMR can rank each feature according to both its dependency to the target classification variable and the redundancy between features. Evaluating performance of three different sequence-encoding schemes, including AA (amino acid sequence encoding), AA + SS + SA + DO (amino acid sequence + secondary structure + solvent accessibility + native disorder, without feature selection), and mRMR (mRMR feature selection based on all the extracted initial features) allowed us to assess the individual contributions of various major types of features to model performance and the importance of feature selection. Figure 3 contains the ROC curves of three different sequence-encoding schemes for four kinase families, including CDKs, MAPKs, PKC, and CK2. These data were the result of 5-fold cross-validation tests using the benchmark datasets.

Performance of RF-based models improved for all four kinase families following mRMR feature selection. Specifically, the models trained by mRMR using the selected feature set achieved an AUC score of 0.991, 0.981, 0.953, and 0.975 for the four kinase families, respectively, outperforming the models trained using the other two sequence-encoding schemes. In addition, Table 3 contains the values of the eight performance measures for all 12 kinase families. These results show that performance of the model trained using mRMR-selected features was the best among the three different sequence-encoding schemes. This was the case for all 12 kinase families, except the PKA kinase family, for which the performance of the mRMR feature-based model was slightly lower than that of the AA feature-based model (Table 3).

**Table 3:** Performance comparison with different sequence encoding schemes based on the 5-fold cross-validation tests.

Kinase family	Encoding scheme	Accuracy (%)	Sensitivity (%)	Specificity (%)	Precision (%)	Recall (%)	F-Score	MCC	AUC
ATM	AA	94.8	96.5	93.1	93.3	96.5	94.9	0.029	0.954
	AA + SS + SA + DO	85.3	82.8	87.9	87.3	82.7	85.0	0.749	0.911
	mRMR	**100**	**100**	**100**	**100**	**100**	**100**	**1.00**	**1.00**
CaM	AA	78.9	80.7	77.2	77.9	80.7	79.3	0.667	0.822
	AA + SS + SA + DO	69.3	68.4	70.2	69.6	68.4	69.0	0.574	0.770
	mRMR	**92.1**	**86.0**	**98.2**	**98.0**	**86.0**	**91.2**	**0.853**	**0.978**
CDKs	AA	94.4	94.2	94.6	94.6	94.2	94.4	0.894	0.962
	AA + SS + SA + DO	91.2	86.7	95.8	95.4	86.7	90.8	0.840	0.973
	mRMR	**96.5**	**95.8**	**97.1**	**97.0**	**95.8**	**96.4**	**0.932**	**0.991**
CK1	AA	59.1	61.4	56.8	58.7	61.4	60.0	0.516	0.560
	AA + SS + SA + DO	68.2	75.0	61.4	66.0	75.0	70.2	0.562	0.685
	mRMR	**87.5**	**77.3**	**97.8**	**97.1**	**77.3**	**86.1**	**0.777**	**0.989**
CK2	AA	82.9	86.5	79.3	80.7	86.5	83.5	0.716	0.898
	AA + SS + SA + DO	79.3	81.2	77.4	78.2	81.2	79.7	0.672	0.884
	mRMR	**92.3**	**90.9**	**93.8**	**93.6**	**90.9**	**92.2**	**0.858**	**0.975**
GRK	AA	54.8	55.6	54.0	54.7	55.6	55.1	0.504	0.595
	AA + SS + SA + DO	77.0	85.7	68.2	73.0	85.7	78.8	0.640	0.768
	mRMR	**92.8**	**88.9**	**96.8**	**96.6**	**88.9**	**92.6**	**0.867**	**0.975**
GSK-3	AA	87.0	88.9	85.2	85.7	88.9	87.3	0.774	0.905
	AA + SS + SA + DO	77.8	87.0	68.5	73.4	87.0	79.7	0.648	0.890
	mRMR	**95.4**	**90.7**	**100**	**100**	**90.7**	**95.1**	**0.911**	**0.984**
MAPKs	AA	87.1	80.8	93.4	92.5	80.8	86.2	0.774	0.911
	AA + SS + SA + DO	83.6	80.8	86.5	85.6	80.8	83.1	0.726	0.922
	mRMR	**94.5**	**93.0**	**96.1**	**96.0**	**93.0**	**94.4**	**0.897**	**0.981**
PKA	AA	**88.8**	**90.4**	**87.2**	**87.6**	**90.4**	**88.9**	**0.800**	**0.932**
	AA + SS + SA + DO	83.2	82.6	83.9	83.7	82.6	83.1	0.721	0.900
	mRMR	88.5	90.4	86.7	87.2	90.4	88.7	0.797	0.931
PKB	AA	89.3	90.4	89.3	89.3	89.3	89.3	0.809	0.889
	AA + SS + SA + DO	77.3	76.0	78.7	78.1	76.0	77.0	0.649	0.878
	mRMR	**96.0**	**92.0**	**100**	**100**	**92.0**	**95.8**	**0.923**	**0.998**
PKC	AA	79.9	83.7	76.1	77.8	83.7	80.6	0.678	0.855
	AA + SS + SA + DO	73.1	74.1	72.1	72.6	74.1	73.4	0.607	0.814
	mRMR	**87.8**	**86.0**	**89.6**	**89.2**	**86.0**	**87.6**	**0.786**	**0.952**

The best results for each kinase and performance measure are highlighted by bold. AA: binary encoding of amino acid sequence; SS: secondary structure; SA: solvent accessibility; DO: disorder; MRMR: sequence encoding scheme after mRMR feature selection based on all features.

### Feature importance analysis

Using the CDK kinase family as an example, the top 50 features ranked by mRMR are provided in Table 4. The AA6_AAseq was ranked first. Previously, amino acid composition surrounding phosphorylation sites was shown to differ significantly between phosphorylation sites and non-phosphorylation sites^30^. Here, using feature selection experiments, we revealed that the sixth residue in the 9-mer sequence was particularly important for model performance. This position may be particularly important for substrate recognition of the kinase.

**Table 4:** The top 50 important features selected by mRMR feature selection for CDKs.

Order	Feature order	Feature type	Score	Order	Feature order	Feature type	Score
1	V107	AA6_AAseq	0.679	26	V3817	Pro_PPI	0.051
2	V2123	Pro_PPI	0.104	27	V198	AA6_SS	0.053
3	V2799	Pro_PPI	0.072	28	V561	Pro_CC	0.052
4	V272	AA1_DISO	0.101	29	V276	AA2_DISO	0.056
5	V3880	Pro_PPI	0.116	30	V5329	Pro_PPI	0.056
6	V1823	Pro_PPI	0.080	31	V5349	Pro_PPI	0.053
7	V5183	Pro_PPI	0.083	32	V1806	Pro_PPI	0.050
8	V192	AA4_SS	0.087	33	V5492	Pro_PPI	0.051
9	V4866	Pro_PPI	0.086	34	V288	AA9_DISO	0.052
10	V287	AA9_DISO	0.077	35	V4400	Pro_PPI	0.051
11	V1658	Pro_PPI	0.079	36	V4659	Pro_PPI	0.052
12	V1579	Pro_PPI	0.071	37	V5205	Pro_PPI	0.052
13	V195	AA5_SS	0.070	38	V271	AA1_DISO	0.051
14	V789	Pro_pathway	0.071	39	V2756	Pro_PPI	0.048
15	V1636	Pro_PPI	0.066	40	V2464	Pro_PPI	0.047
16	V277	AA3_DISO	0.064	41	V193	AA5_SS	0.048
17	V4166	Pro_PPI	0.065	42	V4096	Pro_PPI	0.048
18	V5110	Pro_PPI	0.069	43	V546	Pro_CC	0.049
19	V2710	Pro_PPI	0.058	44	V278	AA3_DISO	0.050
20	V5377	Pro_PPI	0.058	45	V3332	Pro_PPI	0.048
21	V285	AA8_DISO	0.058	46	V5064	Pro_PPI	0.046
22	V3429	Pro_PPI	0.056	47	V809	Pro_pathway	0.046
23	V3376	Pro_PPI	0.058	48	V286	AA9_DISO	0.047
24	V4179	Pro_PPI	0.057	49	V4234	Pro_PPI	0.047
25	V183	AA1_SS	0.053	50	V2516	Pro_PPI	0.047

Annotations of feature types: AA*n*_AAseq (V1-V180): Binary encoding amino acid sequence (180-dimensional vector), where *n* (*n* = 1, 2, … 9) denotes the residue position in the local window size of 9 residues. AA*n*_SS (V181-V207): Secondary structure predicted by SABLE (27-dimensional vector); AA*n*_SA (V208-V270): Solvent accessibility predicted by SABLE (63-dimensional vector); AA*n*_DISO (V271-V288): Native disorder predicted by DISOPRED2 (18-dimensional vector); Pro_BP (V289-V536): Over-represented Biological Process features from Gene Ontology (248-dimensional vector); Pro_CC (V537-V587): Over-represented Cellular Component features from Gene Ontology (51-dimensional vector); Pro_InterPro (V588-V774): Over-represented features from InterPro (187-dimensional vector); Pro_pathway (V775-V818): Over-represented pathway features from KEGG (44-dimensional vector); Pro_MF (V819-V895): Over-represented Molecular Function features from Gene Ontology (77-dimensional vector); Pro_domain (V896-V946): Over-represented functional domain features from Pfam (51-dimensional vector); Pro_PPI (V947-V5698): Over-represented protein-protein interactions from PPI (4752-dimensional vector).

Notably, a total of 35 functional features were selected and included in the list, including 31 PPI features (denoted as Pro_PPI), two pathway features (denoted as Pro_pathway), and two CC features (denoted as Pro_CC) (Table 4). Additionally, another important feature group includes native disorder features (denoted as AA#_DISO, where “#” represents 1, …, 9, indicating the residue position in the 9-mer sequence), which includes nine scores. The disorder-score distributions are significantly different between phosphorylation and non-phosphorylation sites, with phosphorylation sites having higher disorder scores on average than non-phosphorylation sites^30^. This implies that phosphorylation sites are preferentially located in disordered regions. This observation is consistent with several previous studies^31, 34^ on kinase-specific phosphorylation site prediction, which also used protein disorder features to train their respective prediction models.

Furthermore, secondary structure information is also an important feature for model performance. There are five features included in the list of the top 50 features, namely AA4 (V192), AA5 (V195), AA5 (V193), AA6 (V198), and AA1 (V183) (Table 4). Our feature selection analysis revealed that the secondary structures of the first, fourth, fifth, and sixth residues in the 9-mer sequence window were more important than secondary structures of other positions. These results suggest that secondary structures associated with these residue positions contribute to recognition and specificity of the CDKs.

### Performance comparison between different tools on the two independent test datasets

To evaluate the performance of kinase-specific phosphorylation site prediction by PhosphoPredict, we compared its results with those of four popular tools, including KinasePhos^23, 24^, PPSP^26^, Musite^30, 31^, and GPS^38–40^. We would like to point out that in practice it is very difficult to rigorously compare the performance of all tools in an objective and non-biased manner. Some of the important guidelines for constructing unbiased and diverse data sets and performing stringent performance comparison studies based on various biologically relevant considerations have been recently discussed^75^.

In this study, all the compared tools were implemented as online webservers or local stand-alone Java programs; in most cases, it is almost impossible to keep up to date with the knowledge of the state-of-the-art training datasets that these webservers or tools have used to train their prediction models, especially after recent major upgrades. Given that most phosphorylation site prediction tools have been trained using data from Phospho.ELM, it would not be a fair comparison if we performed independent tests and evaluated the performance of different tools using the extracted data from the same resource. Therefore, to make a fair performance comparison, we prepared two independent test datasets, termed as “PhosPlus_set” and “UniProt_set”. The performance results were generated by directly submitting the sequences to their respective webservers or stand-alone programs and retrieving their prediction outputs. For the Phosplus_set, we could not extract sufficient independent test data for the MAPKs and as a result we only performed independent tests for the four kinases CDKs, CK2, PKA, and PKC. Performance comparisons for PhosPlus_set and UniProt_set are provided in Tables 5 and 6, respectively.

**Table 5:** Performance comparison of several prediction tools based on the PhosPlus_set.

Kinase	Method	Accuracy	Sensitivity	Specificity	Precision	Recall	F-Score	MCC	AUC
CDKs	KinasePhos	86.6	65.2	86.9	5.8	65.2	10.6	0.195	0.777
	PPSP	91.0	74.1	91.2	9.4	74.1	16.8	0.261	0.838
	GPS	84.4	**78.0**	84.5	5.8	78.0	10.9	0.206	0.881
	Musite	88.9	77.1	89.0	8.0	77.1	14.4	0.242	0.886
	PhosphoPredict	**94.2**	77.1	**94.4**	**14.5**	77.1	**24.4**	**0.330**	**0.904**
CK2	KinasePhos	89.2	**51.2**	90.0	9.4	**51.2**	16.0	0.229	0.714
	PPSP	93.1	49.4	94.0	14.4	49.4	22.3	0.274	**0.838**
	GPS	94.1	50.0	95.0	17.0	50.0	25.4	0.298	0.821
	Musite	**96.4**	41.6	**97.5**	**25.5**	41.6	**33.1**	**0.331**	0.809
	PhosphoPredict	91.9	50.6	92.8	12.5	50.6	20.1	0.259	0.727
PKA	KinasePhos	90.4	61.6	90.9	11.1	61.6	18.9	0.264	0.775
	PPSP	90.2	73.3	90.5	12.5	73.3	21.3	0.298	0.836
	GPS	85.3	80.1	85.4	8.9	80.1	16.0	0.256	0.880
	Musite	88.9	70.4	89.2	10.8	70.4	18.7	0.273	0.877
	PhosphoPredict	**91.1**	**80.5**	**91.3**	**14.0**	**80.5**	**32.7**	**0.327**	**0.896**
PKC	KinasePhos	81.8	49.4	82.3	4.0	49.4	7.4	0.155	0.677
	PPSP	83.8	**58.8**	84.2	5.3	**58.8**	9.7	0.183	0.734
	GPS	82.1	56.8	82.7	6.6	56.8	11.8	**0.203**	0.785
	Musite	86.7	52.3	87.2	5.8	52.3	10.4	0.183	0.798
	PhosphoPredict	**87.8**	57.2	**88.3**	**6.8**	57.2	**12.2**	**0.203**	**0.826**

The best results for each kinase and performance measure are highlighted in bold.

**Table 6:** Performance comparison of several prediction tools based on the UniProt_set.

Kinase	Method	Accuracy	Sensitivity	Specificity	Precision	F-Score	MCC	AUC
CDKs	KinasePhos	**97.3**	26.5	**98.6**	**26.3**	26.4	0.250	0.626
	GPS	95.6	57.8	96.3	22.7	**32.6**	**0.344**	0.771
	Musite	93.2	**73.4**	93.6	18.1	29.0	0.342	0.841
	PhosphoPredict	93.4	66.7	93.9	17.2	27.3	0.316	**0.857**
CK2	KinasePhos	**96.2**	22.5	**98.2**	**25.3**	23.8	0.219	0.604
	GPS	91.9	**59.6**	92.7	18.1	**27.7**	**0.298**	**0.772**
	Musite	92.6	4.8	95.0	2.5	3.3	-0.002	0.499
	PhosphoPredict	92.5	33.9	94.1	13.5	19.3	0.181	0.712
MAPKs	KinasePhos	**95.0**	40.6	**96.3**	20.7	27.4	0.267	0.687
	GPS	94.7	51.9	95.7	**21.7**	**30.6**	0.313	0.741
	Musite	92.7	**67.2**	93.3	19.6	30.4	**0.337**	0**.816**
	PhosphoPredict	91.0	65.1	91.6	15.1	24.5	0.284	0.810
PKA	KinasePhos	**97.2**	37.7	**98.3**	**28.4**	32.4	0.313	0.682
	GPS	96.8	55.7	97.5	28.0	**37.3**	**0.381**	0.770
	Musite	94.3	**65.1**	94.8	18.0	28.1	0.322	0.808
	PhosphoPredict	95.8	48.3	96.7	20.2	28.3	0.295	**0.845**
PKC	KinasePhos	**96.4**	15.1	**98.3**	17.0	16.0	0.142	0.568
	GPS	95.8	35.8	97.1	**22.5**	**27.6**	**0.263**	0.666
	Musite	93.1	**41.5**	94.2	14.2	21.2	0.214	0.682
	PhosphoPredict	93.3	29.2	94.8	11.4	16.4	0.153	**0.714**

The best results for each kinase and performance measure are highlighted in bold.

GPS is a method developed using a group-based phosphorylation scoring algorithm and is regarded as a sequence similarity-based clustering approach^38–40^. Compared with machine-learning methods, GPS is simpler and faster and constitutes a kinase-specific phosphorylation site prediction method. When evaluated on the PhosPlus_set, GPS achieved AUC scores of 0.881, 0.821, 0.880, and 0.785 on the PhosPlus_set for CDKs, CK2, PKA, and PKC families, respectively (Table 5), while on the UniProt_set it achieved AUC scores of 0.771, 0.772, 0.741, 0.770 and 0.666 for CDKs, CK2, MAPKs, PKA, and PKC, respectively (Table 6).

Musite is a tool used for both general and kinase-specific phosphorylation site prediction^30^ and utilizes datasets from different databases, such as Phospho.ELM, PhosPhAt^76^, and UniProt, to train SVM classifiers. On the PhosPlus_set, Musite achieved AUC values of 0.886, 0.809, 0.877, and 0.798 for CDKs, CK2, PKA, and PKC families, respectively (Table 5). While on the UniProt_set, Musite achieved AUC values of 0.841, 0.499, 0.816, 0.808 and 0.682 for CDKs, CK2, MAPKs, PKA, and PKC, respectively (Table 6).

PPSP is a webserver based on Bayesian decision theory^26^ and the models were trained using datasets extracted from Phospho.ELM. PPSP attained AUC values of 0.838, 0.838, 0.836, and 0.734 on the PhosPlus_set for CDKs, CK2, PKA, and PKC families, respectively (Table 5). In particular, the AUC of PPSP for MAPKs was the highest among all four tools. Note that at the time of performing the performance comparisons based on the UniProt_set, PPSP was inaccessible and thus its performance was not included in Table 6.

KinasePhos is a webserver based on hidden Markov models and is capable of identifying kinase-specific phosphorylation sites^23, 24^. The datasets used by KinasePhos were extracted from PhosphoBase and Swiss-Prot. On the independent datasets, the AUC values of KinasePhos on the PhosPlus_set were 0.777, 0.714, 0.775, and 0.677 for CDKs, CK2, PKA, and PKC families, respectively (Table 5). While on the UniProt_set, KinasePhos achieved AUC values of 0.626, 0.604, 0.687, 0.682 and 0.568 for CDKs, CK2, MAPKs, PKA, and PKC, respectively (Table 6).

**Figure 4:**
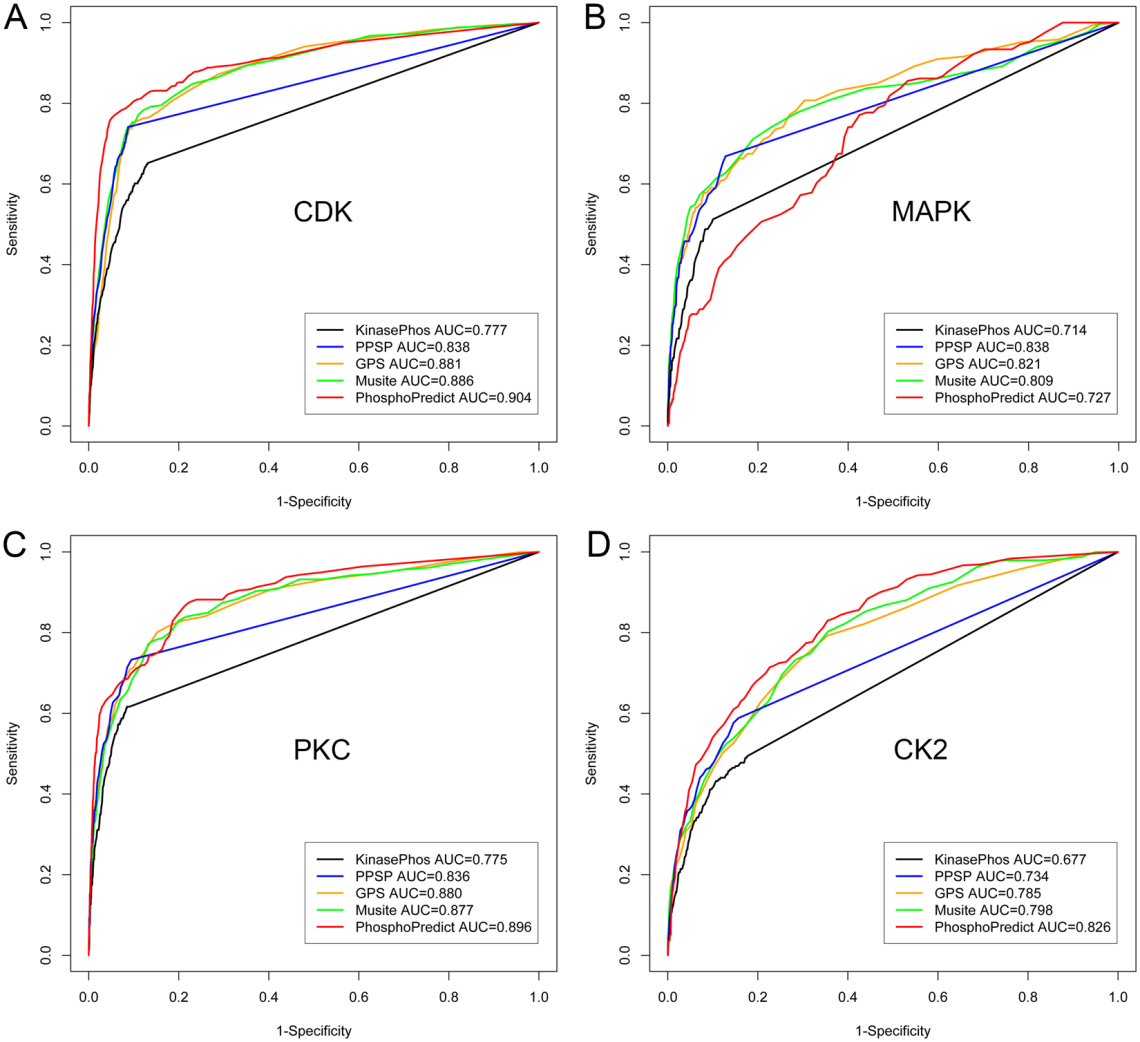
Comparative phosphorylation site prediction. ROC curves for kinase-specific phosphorylation site prediction between PhosphoPredict and the four currently-available tools, including KinasePhos, PPSP, GPS, and Musite. Data represent (**A**) CDKs, (**B**) MAPKs, (**C**) PKC, and (**D**) CK2.

Compared with these four tools, our method PhosphoPredict achieved the performance (AUC) of 0.904, 0.727, 0.896, and 0.826 on the PhosPlus_set_ for CDKs, CK2, PKA, and PKC families, respectively (Fig. 4 and Table 5). PhosphoPredict achieved the highest AUC scores for three kinase families (CDKs, PKA, and PKC), with the only exception being CK2, for which its performance lagged behind that of PPSP, GPS, and Musite, but was better than that of KinasePhos. Other performance measures, such as ACC and MCC, saw similar trends. On the UniProt_set, PhosphoPredict achieved the highest AUC values of 0.857, 0.845 and 0.714, for CDKs, PKA, and PKC, respectively, while for the other two kinase families, CK2 and MAPKs, it achieved the second highest AUC values. In summary, PhosphoPredict performed comparably to or better than the other four tools on both independent test datasets.

### Proteome-wide prediction analysis of potential phosphorylation sites in the human proteome

The most important advantage of computational methods as compared to experimental methods is the ability to efficiently screen unknown or uncharacterized phosphorylation sites, saving both time and cost. PhosphoPredict was used to screen the entire human proteome, consisting of 81,194 proteins, for potential phosphorylation sites for all 12 kinase families (Table 7), using a specificity level of 99%. Corresponding results for the entire human proteome can be freely downloaded at http://phosphopredict.erc.monash.edu/. Our predictions of phosphorylation sites provide valuable hypotheses to be experimentally validated.

**Table 7:** Proteome-wide kinase-specific phosphorylation site predictions.

Kinase	Number of predicted phosphorylated substrates	Number of predicted phosphorylation sites
ATM	153	737
CaM	194	402
CDKs	734	3786
CK1	202	673
CK2	329	809
GRK	166	388
GSK-3	136	339
MAPKs	812	4365
PKA	488	889
PKB	491	1220
PKC	152	325
SRC	315	542

Predictions used a cutoff value of 0.8, which corresponded to a specificity of 99%. Prediction was performed for the whole human proteome with a total of 81,194 proteins. Results are available for download at http://phosphopredict.erc.monash.edu/.

### Functional enrichment analysis of predicted kinase-specific substrates in the human proteome

**Figure 5:**
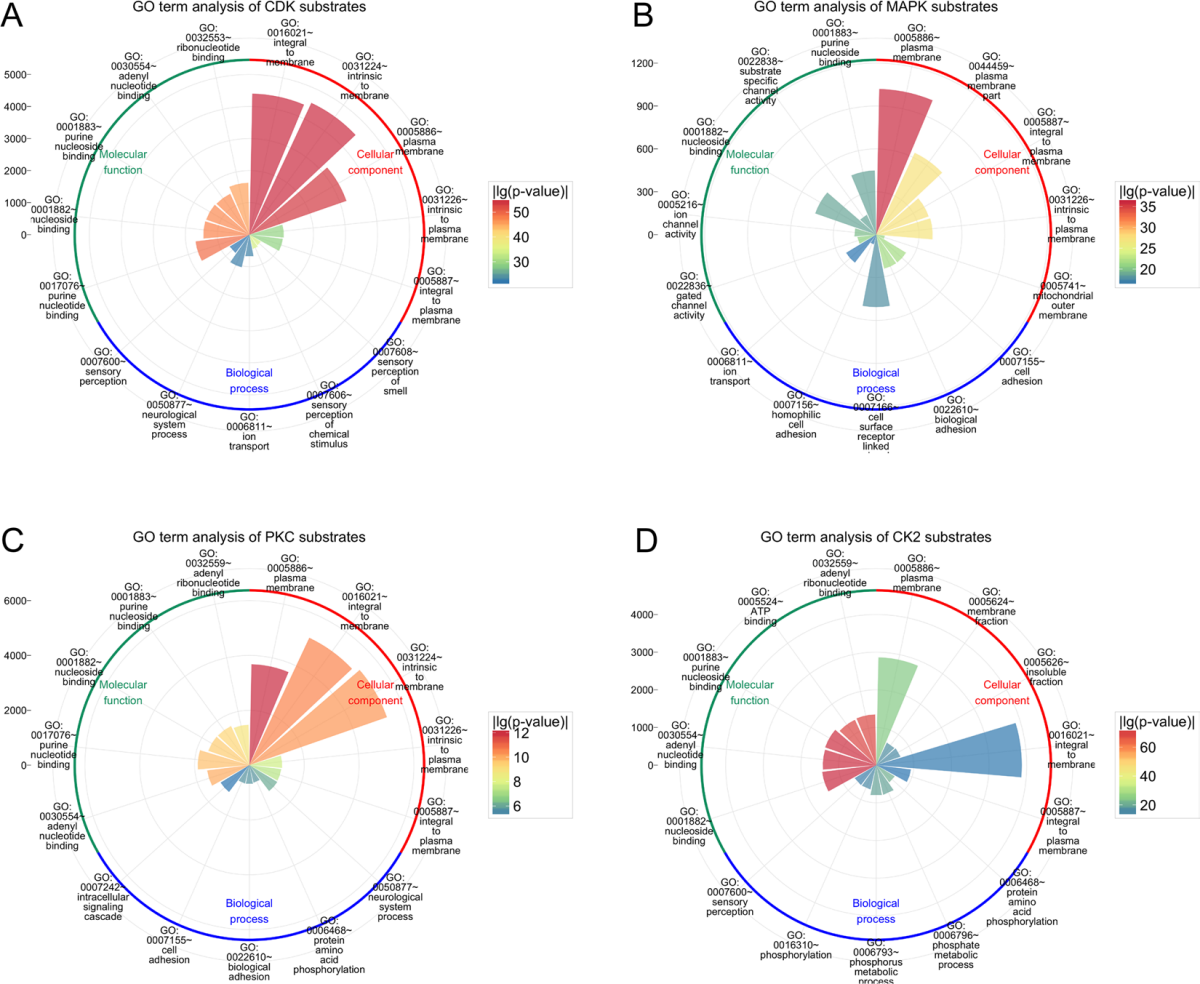
Functional enrichment analysis of the predicted substrates of four different kinases at the proteome level, in terms of three major categories, i.e. cellular component (GO_CC), biological process (GO_BP) and molecular function (GO_MF). For each GO category, the top five significantly enriched GO_CC, GO_BP and GO_MF terms are displayed. (**A**) CDKs; (**B**) MAPKs; (**C**) PKC, and (**D**) CK2.

To elucidate the overall functional characteristics, cellular components and biological processes, we further performed a gene ontology (GO) enrichment analysis for the predicted kinase-specific substrates at the proteome level using the DAVID software^77^. In Fig. 5, the sectorial area for a GO term represents the number of proteins of this term while the different color of the sectorial area indicates the statistical significance of the enrichment for the corresponding GO term. Only the top five most enriched GO terms for the four kinases CDKs, MAPKs, PKC and CK2 are displayed in Fig. 5.

Phosphorylated substrates of different kinases are commonly located in the membrane regions (e.g. integral to membrane, intrinsic to membrane, plasma membrane and mitochondrial outer membrane). We also show that phosphorylated substrates are present in diverse cellular processes and pathways, including intracellular signaling cascades, cell surface receptor-linked processes, ion transport, cell adhesion, and sensory perception (Fig. 5). For the CDK substrates, we found that the most significantly enriched GO CC terms are “integral to membrane” (with *p*-value = 9.12e-56) and “intrinsic to membrane” (with *p*-value = 1.33e-55), while for the MAPK substrates, the most significantly enriched terms are “plasma membrane” (with *p*-value = 9.12e-37) and “plasma membrane part” (with *p*-value = 4.10e-28).

In terms of GO Molecular Function, the most enriched GO terms for phosphorylated substrates are associated with nucleoside binding, including adenyl nucleotide binding, purine nucleotide binding and ribonucleotide binding. Indeed, recent studies show that nucleotide-binding protein substrates can be targeted and regulated by multiple kinases such as CDKs, MAPKs, PKA and PKC^78^. In particular, we also show that phosphorylated MAPK substrates are significantly enriched for gated channel activity (with *p*-value = 2.241e-21) and ion channel activity (with *p*-value = 1.961e-20).

Moreover, we also observe some interesting differences in the significantly enriched GO terms between different kinase substrates from Fig. 5. For example, MAPKs and PKC are especially enriched in specific GO terms compared to the other two kinases CDKs and CK2, and the presence of adhesion/cell surface receptor linked/intracellular signalling cascade are consistent with the known functional roles for MAPKs and PKC^79^. In addition, plasma membrane-associated substrates are enriched for CDKs, which may reflect non-canonical roles beyond cell cycle regulation^80^. Altogether, the functional enrichment analysis of predicted kinase-specific substrates in this section sheds light on the functional commonality and diversity of the potential repertoires of these kinase families.

### Availability of the Java program, PhosphoPredict

**Figure 6:**
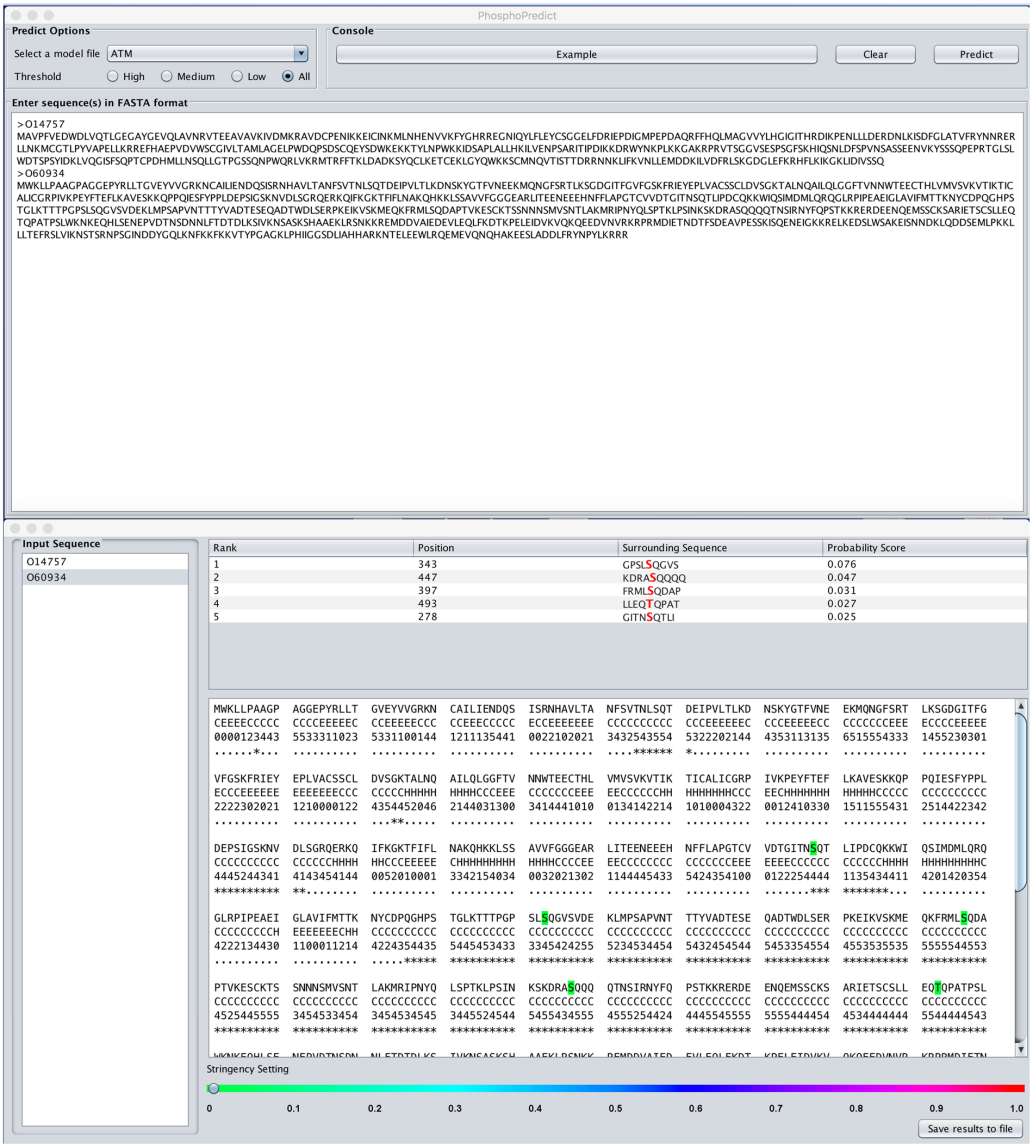
Example output of the PhosphoPredict Java application. Predicted phosphorylation sites of the cell cycle regulatory protein p95 (Nibrin, Uniprot ID: O60934) by the ATM kinase are displayed.

A user-friendly Java version of PhosphoPredict has been developed and implemented with an easy-to-use interface, which can be downloaded from http://phosphopredict.erc.monash.edu/. This program was configured on a 16-core server with 50 GB memory and a 4 TB hard disk. It can be executed on different operating systems, including Windows, Mac OS X, and Linux. Users are required to select the kinase model of interest from a dropdown menu, paste the amino acid sequences of the query protein (in FASTA format), choose the prediction threshold, and then click the “predict” button. An example of the prediction output is provided (Fig. 6). Nbs1 is a component of the MRN complex which plays a critical role in the cellular response to DNA damage and is phosphorylated by the ATM kinase on two sites S278 and S343 in response to radiation damage^81^. As can be seen from Fig. 6, PhosphoPredict correctly predicted the two well-characterized phosphorylation sites and potentially other sites (S397, S447, and T493).

In terms of prediction output display, there are two main sections of the prediction output, including the section of 9-mer sequence ranking and a summary of the secondary structure, solvent accessibility, and disordered region of the submitted sequence, as well as predicted phosphorylation sites highlighted by different colors (corresponding to the predicted probability score). It should be noted that the local Java program and the online webserver of PhosphoPredict differ in the way prediction results are presented. Moreover, the server output webpage provides users with an additional feature: when hovering the mouse cursor over the “?” icon, which is next to each result section headers (original sequence, native disorder, secondary structure and solvent accessibility), a window pops up displaying additional information about the associated result section (See Figure S2 for an example). In addition, the computational time required for a prediction depends on the length of the submitted sequence. For a protein sequence consisting of 500 amino acids, the prediction task requires approximately two minutes to generate and return prediction results. Additionally, PhosphoPredict allows adjustment of the prediction threshold to meet different requirements and results to be saved as a txt (.txt) file for further analysis.

Our PhosphoPredict Java program has been tested on several operating systems, including Windows, Linux and Mac OS X. We highlight that, to run our software in Windows, Mac OS X and Linux systems, users should make sure they have installed and configured the Java JDK1.8 (or newer) on their local computer(s). To that effect, users are advised to download the proper JDK package from http:/www.oracle.com.

### Limitations and future work for developing improved algorithms

Although our approach improves the prediction of phosphorylation sites for several kinases, it has certain limitations. Interestingly, while the inclusion of additional features improved the prediction accuracy for some kinases/kinase families (e.g. CK1 and GRK) it decreased the performance for others (e.g. PKA, PKB, and PKC) (Table 3). The underlying reasons for this observation are not evident but might be associated with the size of the datasets. In addition, incorporation of additional features can also lead to the inclusion of unwanted noisy and/or irrelevant features, which in turn might lead to a performance decrease, if exercised without applying any proper feature selection procedures. Indeed, as can be observed from Table 3, after performing mRMR feature selection, the model performance increased significantly for all the kinases except PKA. This highlights the necessity and value of applying feature selection to heterogeneous feature sets in order to improve the model performance.

On the other hand, PhosphoPredict does not consider other potentially relevant features, such as those with functional context, e.g. surrounding contexts including cell cycle progression, prior phosphorylation events, and determinants of kinase-substrate phosphorylation at the network level^82^. Incorporating such context data and thus complementing the given sequence information, may well improve the accuracy of prediction models and help reduce high false positive rates. In this context, inclusion of informative features (e.g. amino acid property descriptors from the Amino Acid Index Database^83^) that have previously proven useful in other protein bioinformatics studies^84^ may also be helpful for improving the prediction performance of kinase-specific phosphorylation substrates and sites. In this regard, a variety of common features used in previous studies are useful for phosphorylation site prediction, which include local amino acid sequences surrounding potential phosphorylation sites in terms of binary encoding scheme^35^ or amino acid frequency^30, 31, 34, 51, 61^, protein secondary structure^34^, native disorder^34^, and functional features in the form of GO terms^34^ and protein-protein interactions^33–35, 82^. In future work, it will be of particular interest to identify novel contributing features, which can be used in combination to further improve the prediction performance. Lastly, it remains a challenging task to assign reliable negative data, i.e. sites that cannot be phosphorylated under any conditions. In this regard, by combining sequence information with functional context data, the positive-unlabeled (PU) learning technique^85^ might represent a useful framework for building accurate models and reducing the bias caused by selection of negative samples. These and other approaches addressing the limitations of our current method will likely lead to the development of next-generation algorithms with improved phosphorylation site prediction.

## Conclusion

Identifying protein phosphorylation sites is a crucial step in understanding regulatory functions in biological systems. Computational approaches are cheaper, less time consuming, and more practical and efficient for large-scale prediction of phosphorylation sites, as compared with experimental methods. Here, we have developed a new bioinformatics tool, PhosphoPredict, specifically designed for large-scale prediction of phosphorylation sites. PhosphoPredict treats phosphorylation site prediction as a binary classification problem and uses an RF-based machine-learning approach to solve it. Furthermore, PhosphoPredict incorporates both sequence-derived and functional features for kinase-specific prediction of substrates and phosphorylation sites, here applied to 12 kinase families while using mRMR feature selection to significantly improve performance. Benchmarking experiments indicate that PhosphoPredict provides a predictive performance that is competitive with or even superior to four currently available tools. Moreover, the techniques and framework used by PhosphoPredict are applicable to other prediction problems involving protein PTMs, such as acetylation, ubiquitination, sumoylation, methylation and glycosylation. It is our expectation that the PhosphoPredict program and the developed framework described in this study are useful and widely applicable for facilitating accurate prediction and functional annotation of post-translationally modified substrates and sites in the human proteome.

## Electronic supplementary material

Supplementary Figures

Supplementary Table S1
