# Supplementary material for: PhosphoPredict: A bioinformatics tool for prediction of human kinase-specific phosphorylation substrates and sites by integrating heterogeneous feature selection
Source: Sci Rep. 2017 Jul 31;7:6862. doi: 10.1038/s41598-017-07199-4 (PMC5537252; doi:10.1038/s41598-017-07199-4)
Supplement: Supplementary file 1 — Supplementary Figures [file 41598_2017_7199_MOESM1_ESM.pdf]

PhosphoPredict: A bioinformatics tool for prediction of human kinase-specific phosphorylation substrates and sites by integrating heterogeneous feature selection

Jiangning Song, Huilin Wang, Jiawei Wang, Andre Leier, Tatiana Marquez-Lago, Bingjiao Yang, Ziding Zhang, Tatsuya Akutsu, Geoffrey I. Webb and Roger J. Daly

Supplementary Information

Supplementary Figure S1. Sequence logos generated using pLogo for ERK, p38 and JNK MAPKs, and the overall consensus motif for the MAPK family.

Supplementary Figure S2. Example of output webpage from the online web server of PhosphoPredict.

Supplementary Table S1. Prediction performance of different sequence encoding schemes based on the 5-fold cross-validation tests.

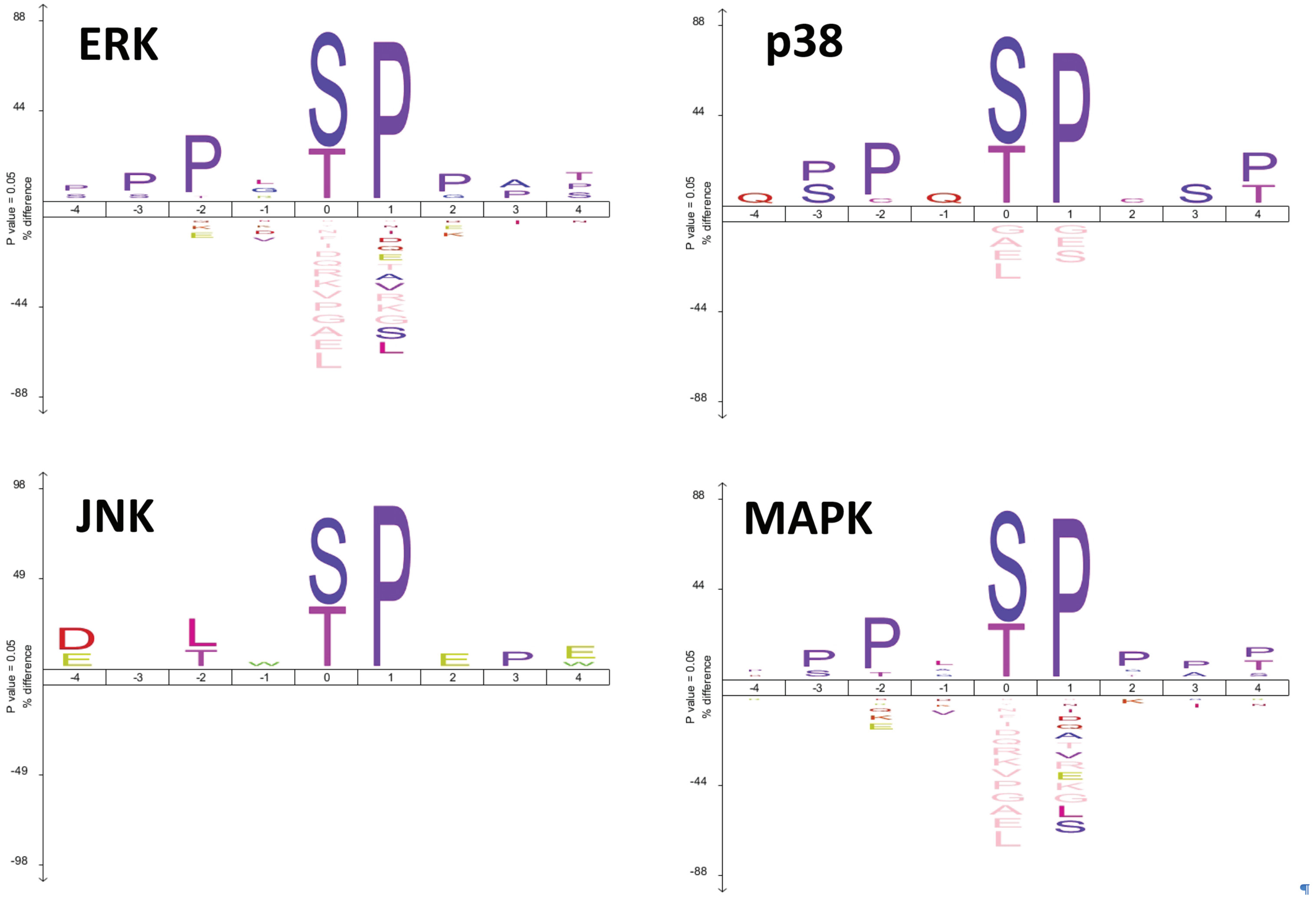

Supplementary Figure S1. Sequence logos generated using pLogo for ERK, p38 and JNK MAPKs, and the overall consensus motif for the MAPK family.

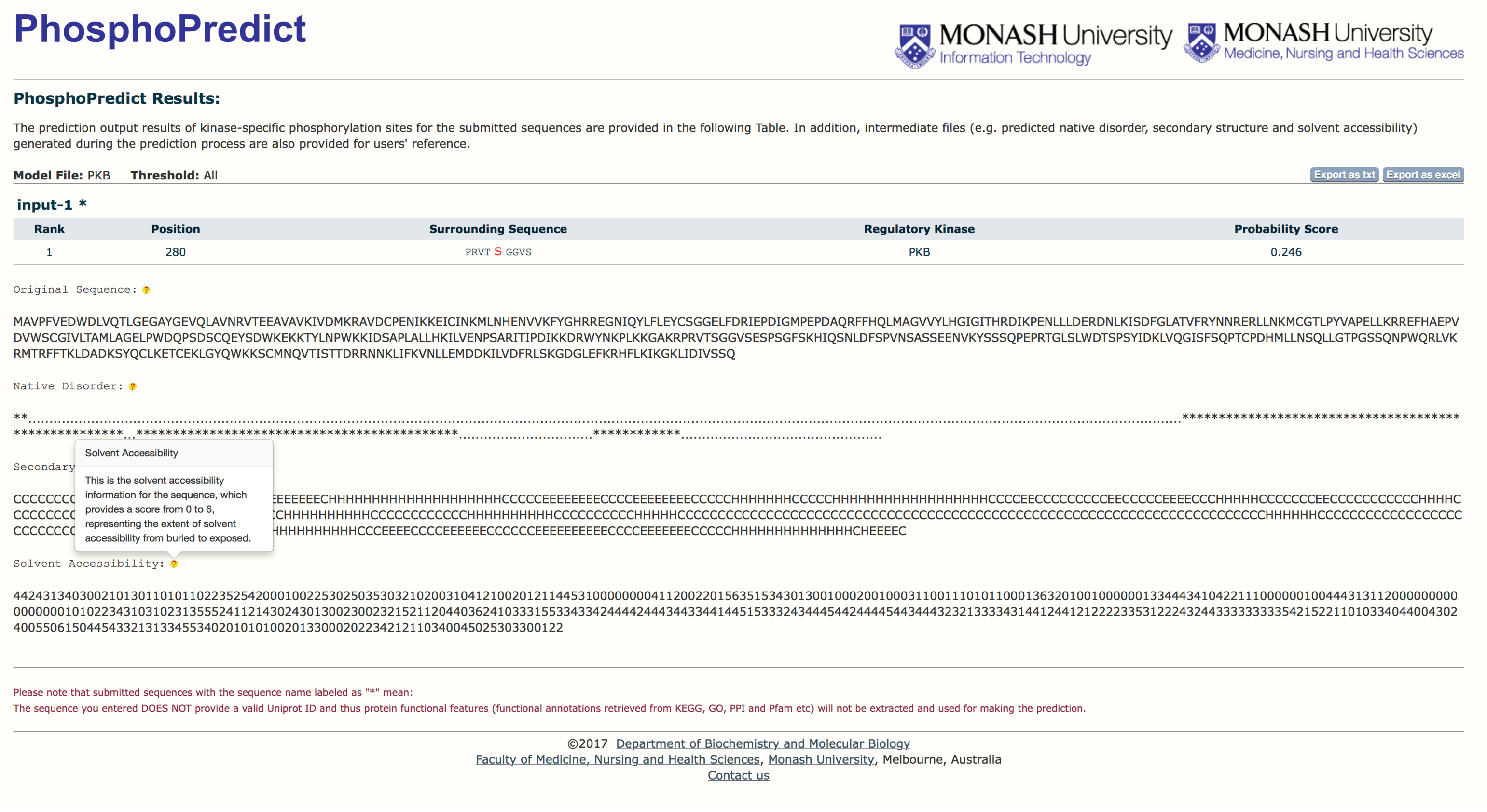

Supplementary Figure S2. Example of output webpage from the online web server of PhosphoPredict.
